# Safeguarding youth from agricultural injury and illness: The Australian experience

**DOI:** 10.3389/fpubh.2022.1059297

**Published:** 2023-01-04

**Authors:** Richard C. Franklin

**Affiliations:** Public Health and Tropical Medicine, College of Public Health, Medical and Veterinary Sciences (CPHMVS), James Cook University, Townsville, QLD, Australia

**Keywords:** children, Australia, injury, safety, home, death, farm

## 1. Background

As of 31 March 2022, there were 25,890,773 estimated resident population in Australia across the eight states and territories (1). In the 2020–21 Financial year, there was 387 million hectares of agricultural land (50% of Australia's land mass), of which the majority (86%) was used for grazing (2). There were 228,372 people in the Australian agricultural workforce in 2016, 69% were males, the median age was 56 years, and a third (37%) were owner operators (3).

In Australia, fatalities on Australian farms have remained steady over the last decade, with 1,584 between 2001–2020 (4). Children represent approximately 15% of these deaths (5, 6). Issues that have been found to be of importance to farmers about the safety of their children on farms in Australia include: general danger avoidance and safety, machinery, moving vehicles, bike safety, animal handling, personal protective equipment (PPE), supervision, speed, water safety and chemicals (5).

Peachey et al. (2020) explored the 222 deaths of children (< 15 years) on Australian farms over a 19-year period (2001–2019) (6). Of the 222 deaths, 51% were less than 5 years of age, 22% were 5–9 years and 27% were 10–14 years (6). The majority (68%) of these deaths were residents of the farms (6). In the final years (2017–2019) of the study there were 13.0 cases per annum, a significant reduction from 1989–1992 where there were 29 deaths per annum (6, 7). Common agents involved in child deaths have not changed much over time, with the exception of quadbike-related deaths of which there were four between 1989–1992 and 32 in the 2001–2019 period, with drowning in dams continuing to be a major cause of death (6, 7). Interestingly, while water safety accounts for 31% of deaths on farms this was not seen as the highest priority by parents (5, 6). Farm dams remain an ongoing challenge for farm safety (8, 9), partly due to changing climatic conditions with dam water level ranging dramatically from year to year. Other common agents, also reflected in the concerns of parents included quadbikes, tractors, utes (farm pick-up), cars, motorcycles and horses (6).

The leading organization responsible for improving safety on farms in Australia is Farmsafe Australia (FSA). FSA is a not-for-profit organization dedicated to improving the wellbeing and productivity of Australian agriculture through enhancing health and safety practices. FSA grew from local action in the 1980's and has operated as Australia's leading agricultural health and safety organization. FSA develops and delivers a range of resources and programs in Australia and has recently (2019) received multi-year funding from the Australian Government to enhance farm safety (10). Recent activity from FSA has included the production of community service announcements, reports, refreshed guides and brochures, data collection around safety issues, conferences, and attendance at field days (www.farmsafe.org.au).

Toward the end of the 2000's and into the 2010's a number of research activities around child safety reached their culmination, this included work around developing an evidence base for child injury prevention priorities for Australian farms (11), safe play areas (9, 12), water safety (8, 9, 13), and links to the Australian curriculum (14). A recent literature review (15) identified 41 peer reviewed papers exploring child safety on farms, the review noted that there is limited literature on child safety on farms, that factors which contribute to child injuries on farms include; exposure to hazards, risk-taking, lack of supervisions, children working, lack of regulations including understanding of the hierarchy of control, lack of supervision, financial challenges and poor uptake of safe play areas (15). Unfortunately, there is limited information about the relationship between injuries and work for children on farms in Australia, an area requiring further research.

There is no doubt that child safety on farms represents a “wicked problem” (16), that is a problem that requires multifactorial solutions due to its complexity. To address child injury on farms, FSA has used the hierarchy of control approach [i.e., removing the hazard, substituting for a lesser hazard, engineering solutions, management solutions and lastly personal protective equipment (17)] and this is then reflected in the material they produce and the way safety is communicated.

## 2. Current activities

There has been a wide range of resources developed for child safety on farms in Australia over the years, the following is a summary of those that are still available or known to the Author. Farmsafe Australia has played a significant role in the development of these resources. However, other organizations such as Kidsafe, Royal Life Saving Society – Australia and doctor groups have also been involved. These resources have ranged from practical advice such as the “Child Safety on Farms: A Practical Guide” (18) to pamphlets, brochures, television shows and commercials.

An early example of an integrated child safety program was Giddy Goanna. This program originated in Queensland and went Australia-wide. It included books, activities, television program, a mascot (Giddy Goanna – which was hot to wear) and addressed all farm activities from working with sheep and cattle to chemicals, farm machinery, water safety and feeding pets. The resources as still found in schools and are available to purchase (at Dec-2022) and includes merchandise.

Recently FSA has partnered with “George the Farmer” (https://www.georgethefarmer.com.au) to produce a song with a video about child safety and an educator's guide (19). George the Farmer was developed as a tool for educating children about where their food and fiber come from and is used in schools as an education tool *via* videos and booklets. The educator's guide links to the Australian Curriculum for Year 4 in science, technologies, English, health and physical education (19), however, can also be used with younger children. It provides safety information around jobs and activities on farms and has activities to help embed the learning outcomes.

This work builds on the child safety on farm – a practical guide (18) material and the RIPPER resources (14). The practical guide provides specific advice on how to prevent drowning, injuries due farm motorbike, farm vehicles, horses, tractors and machinery and more general advice around other hazards, it also include a section on “Safe Play Areas” (18). The RIPPER material provides information about farm safety for teachers, links to the curriculum and has a wide range of activity sheets (14). Both of these are still widely used across Australia.

A significant activity of Farmsafe and its partners has been the promotion of safe play areas. This concept originally develop in the USA, was then adopted in Australia with a range of resources to promote the concept to Australian farmers (20). It is especially relevant to farms with young children, as they have been found to wander far from the farm house and end up drowning in dams (8). The program includes information about the benefits of safe play areas', how to make an effective barrier and safe play area, and the role of supervision (20). This work has meant that the number of farms with safe play areas has increased, although only two-thirds of parents reported that it would be secure enough to prevent children from wandering away (12).

Other activities include “Farm Safety Days for Children”, these activities normally take children onto farms and have a range of safety talks, experiential learning, chats about farming and practical advice and involve the local hospital/doctors, Farmsafe, Kidsafe, Royal Life Saving, schools, farmers, stock and station agents, machinery dealers and retailers, parents, and other interested parties. Farmsafe also includes strategies within other areas, for example quadbikes where children are expressly mentioned (21).

Kidsafe (https://kidsafe.com.au) and the Royal Life Saving Society – Australia (https://www.royallifesaving.com.au/) have also developed a range of resources to help parents keep their children on farms safe. This includes videos, books, brochures, guides (https://www.kidsafewa.com.au/resources/) and a sign about shutting the gate (https://www.royallifesaving.com.au/stay-safe-active/locations/farm-water-safety). This material is used widely as part of their community outreach. The Kidsafe parent guide is in its third edition and includes information around how to prevent injuries, safe play areas, specific hazards and what to do in an emergency.

Finally, we should not forget legislative approaches. In Australia, there are two significant areas of legislation, workplace health and safety legislation and child employment legislation. Workplace health and safety legislation takes a risk management approach using the hierarchy of control, however, the child needs to be old enough to be working for this legislation to be relevant. The child employment legislation covers how old, when and what activities children can undertake (22). While these laws are state and territory specific, the general approach is that children should not be working full-time until they are older than 15 years, with some work allowed under this age, however, it should not interfere with school (i.e., should not be during school hours, or be of such a volume that it would impact on learning with some states specifying times when children cannot work), nor place the child in danger with some states specifically banning some types of work (for example dangerous machinery, dangerous substances, working at height, service of alcohol, gambling services, etc.). Often excluded from these provisions is working for family or domestic chores, volunteer work (i.e., unpaid work) or traineeships. This means that work on farms is often excluded from scrutiny under the legislation and thus other strategies to ensure child safety on farms are required (22).

## 3. Impacts and challenges

It is difficult to tell what impact any given resource has had in preventing child injuries and deaths on farms, as there has been very little evaluation of any specific initiative, this is an area that requires further work. Peachey et al. (6) in their exploration of child deaths found that the average number of deaths on farms had decreased from 17.6 deaths per annum in 2001–2003 to 13.0 in 2017–2019, while this is good news overall, unfortunately the death rate remained unchanged representing a decrease in the number of children living on farms (6). Safe play areas have been widely promoted and its uptake has been varied (12) and it is not clear how long the barriers last, noting that it should also be accompanied by a set of rules to guide the child and parents.

Growing up on a farm provides many life experiences that other children do not get to experience, these life experiences are often encouraged by parents, however, there is a need to ensure that they occur safely and that parents understand what they need to do to ensure the safety of their children.

It appears that exposure to hazards continues to be a challenge, especially for children under the age of 5 years (15). With the number of children who are living on farms decreasing and overall smaller family sizes, there is an ongoing need to ensure that new parents are aware of what can be undertaken to ensure the safety of their children. Child care continues to be a challenge for those living and working on farms, especially those away from population centers (23). With a connected world, noting that this is still a challenge for many rural Australians, there is a need to continue to innovate to ensure that safety information is accessible, engaging and valid for the next generation of parents.

## 4. Future directions

While the number of deaths on farms has decreased, mainly due to fewer young children living on farms, little is known about non-fatal injuries of children on farms, and there is a lack of evidence about what works to prevent injuries, both areas that need further exploration. As farms adapt to the changes in legislation [note in 2011, Australia started to update its workplace health and safety legislation to be more consistent (24)] and improve their safety performance, it is hoped that this then flows into the child safety area. This needs to be tested around the impact on farming practices and the safety of children on farms.

The next steps in Australia should be around bringing together all the interested parties in child farm safety and working toward a nationwide coordinated effort, which will hopefully develop a child safety on farms strategy, linked to the hierarchy of control approach, as the guiding principle outlined in workplace health and safety legislation in Australia (25). This would expectantly bring new energy and resources to the cause and thus continue to drive down child deaths and reduce the number of injuries on farms while at the same time ensuring that children continue to develop as young farmers. This will require improving the evidence base for what works, ensuring that researchers, practitioners and policymakers work together and that we continue to develop new and adapt current recourses to the task.

## Author contributions

The author confirms being the sole contributor of this work and has approved it for publication.
